# Reconnecting with nature for sustainability

**DOI:** 10.1007/s11625-018-0542-9

**Published:** 2018-02-28

**Authors:** Christopher D. Ives, David J. Abson, Henrik von Wehrden, Christian Dorninger, Kathleen Klaniecki, Joern Fischer

**Affiliations:** 1 School of Geography, University of Nottingham, University Park, Nottingham, NG7 2RD UK; 2 Faculty of Sustainability, Leuphana University Lüneburg, Universitätsallee 1, 21335 Lüneburg, Germany

**Keywords:** Human–nature relationship, Social–ecological systems, Sustainability, Transformation

## Abstract

Calls for humanity to ‘reconnect to nature’ have grown increasingly louder from both scholars and civil society. Yet, there is relatively little coherence about what reconnecting to nature means, why it should happen and how it can be achieved. We present a conceptual framework to organise existing literature and direct future research on human–nature connections. Five types of connections to nature are identified: material, experiential, cognitive, emotional, and philosophical. These various types have been presented as causes, consequences, or treatments of social and environmental problems. From this conceptual base, we discuss how reconnecting people with nature can function as a treatment for the global environmental crisis. Adopting a social–ecological systems perspective, we draw upon the emerging concept of ‘leverage points’—places in complex systems to intervene to generate change—and explore examples of how actions to reconnect people with nature can help transform society towards sustainability.

## Introduction

Humanity’s relationship to the natural world has been a topic of scholarship since ancient times, yet with growing recognition of environmental crises over the past decades, society’s disconnection from nature has been proposed as a root cause of unsustainability (e.g., Pyle 1993; Folke et al. 2011; Dorninger et al. 2017). Recently, calls for society to ‘reconnect with nature’ have grown louder (Zylstra et al. 2014), with new research emerging in sustainability science, conservation biology, environmental psychology, and environmental education (Nisbet et al. 2009; Folke et al. 2011; Fischer et al. 2012a; Frantz and Mayer 2014). Yet, most calls for ‘reconnection’ have remained speculative and vague, with relatively few concrete insights regarding the characteristics of a connected society or how to achieve this goal. The literature is fragmented across disciplinary boundaries, resulting in low coherence in the ways central concepts are understood and applied (Ives et al. 2017). For example, there is confusion around the concept of connection to nature and whether a state of disconnection is a response to or a driver of social–ecological change, or both. On this basis, it is timely to assess together the disparate strands of scholarship to scrutinise if pursuing an agenda of reconnecting people with nature is worthwhile, and if so, how this aim ought to be pursued.

In this article, we lay a conceptual platform to better understand human–nature connectedness. First, we argue that human–nature connectedness is a multifaceted concept incorporating (1) material connections such as resource extraction and use; (2) experiential connections such as recreational activities in green environments; (3) cognitive connections such as knowledge, beliefs and attitudes; (4) emotional attachments and affective responses; and (5) philosophical perspectives on humanity’s relationship to the natural world. Second, we show that existing literature frames connection to nature as either the cause of some outcome (such as human health or environmentally-responsible behaviour), the consequence of some driver (such as shifting societal values or technological change), or the treatment for social or environmental problems. Finally, having laid this conceptual platform, we outline ways in which people’s connections with nature can be strengthened. We argue that stronger connections—in several of the above-mentioned dimensions—have potential to help leverage deep societal change for sustainability (Meadows 1999; see; Abson et al. 2017). In particular, we discuss the need for ‘reconnection strategies’ that work to change not only the behaviour of individuals, but also address the systemic structures and paradigms that underpin the actions and behaviours contributing to the current global environmental crisis.

## Conceptualising human–nature connections

Many terms related to connections to nature have arisen from various disciplinary schools and normative agendas. One of the earliest concepts is the “biophilia hypothesis” (Wilson 1984), which asserts that humans have an innate desire to connect with nature. The biophilia paradigm underpins much scholarly and practical work to promote interactions with green environments (Kahn and Kellert 2002). “Nature deficit disorder” is a related, more recent concept, which sees children’s reduced contact with outdoor environments as having negative results for their development (Louv 2005). Similarly, “extinction of experience” (Pyle 1993; Soga and Gaston 2016) refers to the phenomenon of urbanisation reducing everyday nature experiences, with implications for health, emotions, attitudes, and behaviour.

From a global sustainability perspective, phrases such as “reconnecting to the biosphere” (Folke et al. 2011), “teleconnections” between local consumption and global land use (Yu et al. 2013) or “telecoupling” of socioeconomic and environmental systems over geographic distance (Liu et al. 2013) are used to emphasise the dependence of human society on natural systems and processes. The literature from a social–ecological systems perspective calls for “recoupling social and ecological systems” (Fischer et al. 2012b) to foster sustainability. Other literature has introduced the term “distance from nature”. Seppelt and Cumming (2016) suggest that humanity must decrease its distance from the natural world in terms of knowledge of contact with nature while increasing ‘distance’ in the sense of direct impacts of human activities on ecosystems to maintain the earth’s life support system.

Similarly, environmental psychologists have amassed a voluminous literature on the concept of “connectedness to nature”, addressing the cognitive and affective domains of individuals’ psyches (see Restall and Conrad 2015 for a review). Key literature from this perspective includes Wesley Schultz’ (2001) work on the notion of “inclusion of nature in self, Mayer and Frantz’s (2004) “Connectedness to Nature Scale”, and Nisbet’s (2009) work on individual “nature relatedness”. These measures typically consider emotional connections, beliefs, and attitudes, and often correlate with other psychological constructs such as value orientations and pro-environmental behaviour (Tam 2013).

The current diversity of approaches to conceptualising and measuring connections with nature has led to a fragmentation of the literature. This is partly due to the term ‘connection’ being applied to qualitatively different concepts. In some instances, connection to nature refers to a cognitive appreciation of being embedded within nature, in others to an emotional attachment, while still others focus on material dependence on nature. Although this diversity of meanings is being addressed by psychologists through ever more expansive psychometric scales of nature connectedness (e.g., Nisbet et al. 2009), these remain focused on the individual scale and cannot integrate society-scale phenomena of connection or disconnection.

**Fig. 1:**
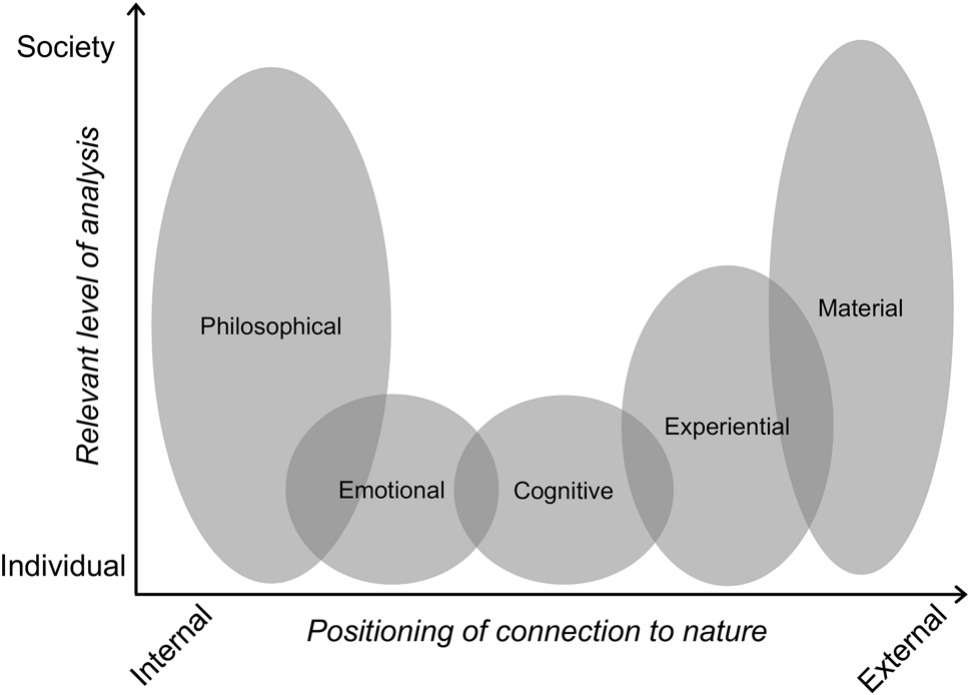
Conceptualisation of different types of human–nature connections, along a spectrum from people’s inner to outer worlds (*x*-axis), and their relevance at different scales of social aggregation (*y*-axis). While presented as independent categories here in this figure, in reality, each type of human–nature connection may interact with the others

In their recent review, Ives et al. (2017) called for more integrated research on human–nature connectedness. To facilitate this and to clarify why and how to reconnect people with nature, we develop our discussion around the five categories of nature connections Ives et al. (2017) proposed: (1) material, (2) experiential, (3) cognitive, (4) emotional, and (5) philosophical connections (Fig. 1.). These can be considered to operate along a spectrum from external connections to nature (e.g., physical appropriation or interaction) through to internal connections to nature (e.g., emotions or worldviews). An additional dimension to consider is the scale at which these connections operate and can be analysed: some connections are understood primarily at the individual scale, while others can be readily aggregated to the societal scale. Descriptions of these dimensions of nature connections are provided in Table 1.

**Table 1:** Descriptions of different types of nature connection

Connection	Description	Analytical scale	Key literature
Material	Consumption of goods/materials from nature (e.g., food, fibre)	Can be analysed for individuals or societies. Often connected to system characteristics. Needs to be spatially explicit (e.g., material flows within or between focal landscapes)	Material flow analysis (Haberl et al. 2004) Human Appropriation of Net Primary Productivity (HANPP) (Haberl et al. 2009) Teleconnections (Yu et al. 2013) Ecological Footprint (Wackernagel et al. 1999)
Experiential	Direct interaction with natural environments (e.g., parks, forests). Note that qualities of connections may vary substantially	Normally measured for individuals, but can be aggregated to the societal scale	Soga and Gaston (2016) Keniger et al. (2013)
Cognitive	Knowledge or awareness of the environment and attitudes/values towards nature	Individual	Bradley et al. (1999) Schultz (2001)
Emotional	Feelings of attachment to or empathy towards nature	Individual	Emotional affinity towards nature scale (Kals et al. 1999) Place attachment to natural areas (Stedman 2003)
Philosophical	Perspective or world view on what nature is, why it matters, and how humans ought to interact with it (e.g., master, participant, steward)	Relevant to individuals, as well as to dominant views at the societal scale	Van den Born (2008) Raymond et al. (2013)

These various dimensions of connection to nature do not operate in isolation—in reality, they interact with and are influenced by one another. For example, physical interactions with natural environments (experiential connections) can shape environmental knowledge and positive attitudes towards the environment (cognitive connections) (Collado et al. 2013). Conversely, people with positive psychological orientations towards nature (emotional and cognitive connections) have been shown to be more likely to visit parks and reserves (experiential connections) (Lin et al. 2014). Ewert et al. (2005) also found that early-life outdoor activities (experiential connections) were related to environmental beliefs (cognitive connections) in adulthood, and Lumber et al. (2017) showed that direct contact with nature along with emotional engagement and contemplation of meaning are associated with a psychological measure of nature relatedness. Many other interactions are likely to exist, but have yet to be examined in depth.

The concept of human–nature connections as outlined above might be considered a theoretical perspective that integrates different relationships between social and natural systems. Other frameworks have been proposed that derive from different applied or theoretical perspectives (see Muhar et al. 2017 for a synthesis of concepts). One of the most commonly applied concepts in environmental management and sustainability is ecosystem services (Millenium Ecosystem Assessment 2003). While related, we consider ecosystem services to be a separate but complementary framework to connection to nature. First, ecosystem services is commonly understood as anthropocentric in focus, since it emphasises the benefits people derive from nature (Schroeter et al. 2014; Silvertown 2015). In contrast, connection to nature is not inherently normative, but describes interactions that may be positive, negative, or benign. Second, ecosystem services have its roots in economic thought, as highlighted by the emphasis on quantifying the ‘value’ of different goods and services that are derived from ecosystems (Silvertown 2015). Human–nature connection represents a broader approach, as highlighted by the ‘philosophical’ dimension which explicitly considers different forms of conceptualising human–nature relationships. Therefore, human–nature connection as a concept is likely to be better positioned to describe and address environmental and sustainability challenges across different socio-cultural contexts.

## Causes, consequences, and treatments

Literature on connection to nature is fragmented beyond differences in the types of connection and scale of analysis. Research also varies according to whether it emphasises (1) the *causes* of nature disconnection, (2) the *consequences* of disconnection, or (3) reconnecting to nature as a *treatment* for some problem. Soga and Gaston (2016) reviewed the literature on the causes and consequences of experiential connections to nature. Yet, similar work to separate causes, consequences, and treatments will be equally important for other dimensions of nature connection.

### Causes of disconnection from nature

Disconnection from nature is often considered as a symptom of broader-scale societal changes (Pyle 2003; Seppelt and Cumming 2016). However, the literature varies according to whether immediate or more fundamental causes of disconnection from nature are considered. Claims about the fundamental causes underpinning disconnection from nature are largely speculative, particularly when considered at the societal scale. Some scholars have argued that disconnection is symptomatic of underlying philosophical or functional shifts such as the dominance of materialism and over-consumption (Pyle 2003). While this may have intuitive appeal, there is little concrete evidence for this assertion. The notion of ‘reconnecting to the biosphere’ proposed by Folke et al. (2011) also implies a historical separation of people from nature, namely, a cognitive disconnection between people’s understanding of the impacts of their activities and biophysical reality. Evidence for such cognitive disconnection is stronger, and can be traced to the increased complexity of global resource systems (see Steffen et al. 2011). Other studies have considered more immediate causes of nature disconnection, and are generally more firmly grounded in empirical evidence. Examples of variables contributing to nature disconnection include urbanisation (Cumming et al. 2014), reduced access to green spaces (Lin et al. 2014), changing social norms and perceptions (Valentine and McKendrck 1997), and rise in electronic media (Pergams and Zaradic 2006).

### Consequences of disconnection from nature

Other studies focus on consequences of being disconnected from nature. Research has spanned fields from child development to sustainability and has addressed matters such as health benefits of outdoor experiences, and individual behaviours associated with emotional or cognitive attachments to nature. One widely publicised consequence of connecting to nature is that of learning and development benefits for children (e.g., Taniguchi et al. 2005). Recent research has pointed to benefits of interactions with natural environments for happiness and general wellbeing (Capaldi et al. 2014) and mental and physical health (Keniger et al. 2013). Furthermore, other literature has demonstrated links between individual nature connectedness and sustainable behaviours (Geng et al. 2015).

At a broader scale, it is commonly asserted in disciplines such as conservation science, environmental psychology, and sustainability science that humanity’s growing disconnection from the natural world is contributing to the global environmental crisis (Nisbet et al. 2009; Zylstra et al. 2014). Kareiva (2008) argued that an experiential separation from nature, as demonstrated through a decline in visitation rates to national parks, “may well be the world’s greatest environmental threat”. While it is difficult to prove empirically that such experiential disconnection poses a threat to biodiversity and sustainability, some evidence has emerged that shows experiences of nature are correlated with willingness to donate to conservation causes (Zaradic et al. 2009) and that psychological connectedness to nature is positively correlated with vegetation protection behaviours by farmers (Gosling and Williams 2010).

### Reconnecting to nature as a treatment

Finally, studies have considered reconnecting people to nature as a treatment, often focused at the individual scale. For example, nature experiences have been explored as treatments for psychological illness such as depression and anxiety (Townsend 2006). Proven health benefits of nature interaction have also led to research modeled on medical approaches such as exploring the nature ‘dose’ necessary to achieve health outcomes (Shanahan et al. 2016). In education, programs that focus on nature experiences as ways of fostering curiosity and resourcefulness are being developed to counteract the dominance of indoor-only play (Mainella et al. 2011). Citizen science has also been explored as a mechanism by which people can connect experientially with nature so as to foster environmental knowledge, concern, and pro-conservation behaviour (Conrad and Hilchey 2011).

Beyond the scale of individuals, a growing body of the literature asserts a need for society to reconnect with nature to facilitate societal transformation towards sustainability (Folke et al. 2011; Abson et al. 2017). Yet, despite the high stakes, nature reconnection as a treatment for society-scale system change has received scant empirical attention to date. We consider that framing human–nature connections as a treatment for social and environmental problems has great merit in the context of myriad challenges facing contemporary society. Yet, researchers must be clear about the motivation for these studies and the mechanisms by which reconnecting people with nature might address the problem at hand, as well as clarifying the overarching narrative they are speaking to (i.e., disconnection from nature as a cause or a symptom).

While some have argued for a reconnection between people and nature, others have called for society to be decoupled from the environment to ensure planetary sustainability. Two aspects of decoupling are often conceptualised: (i) resource decoupling, which denotes a separation of economic activity from resource use, and (ii) impact decoupling, which conceptualises a separation of economic activity from environmental impacts (UNEP 2011). We consider that disconnections from nature and eco-economic decoupling are related, but distinct terms, and are compatible in different contexts. The typology of nature connections we present can help demonstrate this. Reconnection with nature in a cognitive sense might be necessary for a decoupling of economic growth from environmental impacts. Furthermore, issues of scale are critical, since decoupling of economic activity from natural resources almost always conceptualises human–nature connections at the societal scale. By reconnecting people materially to *local* ecosystems and reducing global teleconnections, any impacts to the environment will be recognised more easily, thus decoupling human economic activity from degradation elsewhere.

## Reconnecting people with nature for sustainability?

The preceding sections sought to bring clarity to the multi-dimensionality of concepts and perspectives that characterise the literature on human–nature connections. Specifically, we distinguished five types of nature connections and the societal scales at which they operate, and found that the existing literature can be characterised as framing nature connectedness as a cause, consequence, or treatment to a problem. Here, we explore how reconnecting people with nature can act as a treatment for key sustainability challenges by looking at the five types of nature connectedness from social–ecological systems perspective. Social–ecological systems (or coupled human and natural systems) are complex systems, characterised by multiple interactions and feedbacks between human and natural elements (Fischer et al. 2015). Such a framing is therefore important when addressing sustainability problems, because these problems arise from a complex interplay between environmental and socio-political factors (Fischer et al. 2015). While social–ecological system thinking has been critiqued for subjective definitions of systems boundaries (e.g., Epstein et al. 2013) and under-theorising political and economic dynamics in environmental management (Cote and Nightingale 2012), the framework outlined below provides a useful heuristic way of organising actions for reconnecting people with nature.

### Leverage points

Assuming that “reconnecting” people with nature could be a treatment for the global sustainability crisis, how exactly might an agenda of reconnecting people and nature bring about systemic change? In this section, we draw on the notion of “leverage points” to scrutinise the logic underpinning a possible reconnection agenda. Following Meadows (1999), leverage points are places within complex systems, where interventions can be directed to bring about change in overall system behaviour.

Leverage points can be shallow or deep according to the type of influence they have on a system. Changes to shallow leverage points are relatively ineffective, whereas even minor changes to deep leverage points can alter overall system behaviour. Shallow leverage points relate to (1) system parameters and (2) feedbacks between variables. In contrast, deep leverage points relate to (3) the system design or architecture and (4) the goals or intents pursued through the system. In a sustainability context, this means that changing certain parameters in a system (e.g., the proportion of protected land) is likely to be a less effective leverage point than changing its design (e.g., the rights of biodiversity to persist) or overarching goal (e.g., respect for rather than exploitation of nature). Here, it is important to note that shallow leverage points, such as increasing the amount of protected land, are crucial. However, our ability to increase this parameter is fundamentally constrained by the design of the system and the goals to which the system is oriented. Therefore, focusing only on shallow interventions is unlikely to bring about major changes in system behaviour (Abson et al. 2017).

**Fig. 2:**
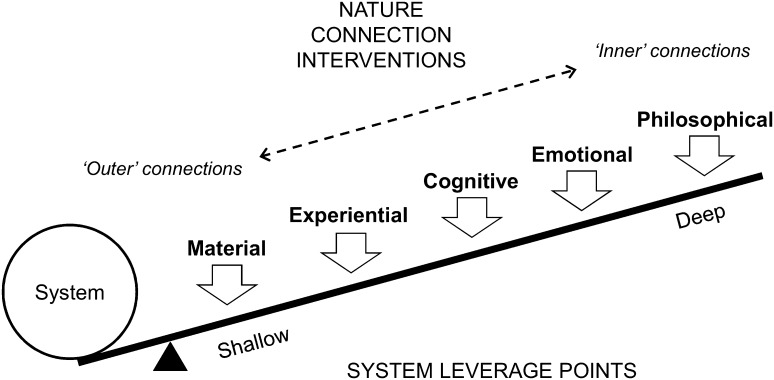
Hypothesised mechanisms by which interventions for reconnecting people with nature can bring about system change. More externally-defined connections to nature (e.g., material and experiential connections) are more likely to influence system parameters (such as resource stocks and flows), while internally-defined connections (such as philosophical perspectives and emotional responses to nature) are more likely to influence the underlying goals and values embodied in a system. We note that connections to nature may affect system properties in more complex ways than are represented here, and system attributes and different types of interventions are likely to interact

This framing around deep versus shallow leverage points provides a working hypothesis regarding how different types of “reconnection” may be more or less effective in fostering sustainability (Fig. 2). Particularly, we propose that connections to nature related to the design or goal implicit in a given system are more likely to have a strong effect on sustainability outcomes than connections related to parameters or feedbacks. It follows that addressing “inner” connections (such as philosophical and cognitive connections) is necessary to bring about sustainability transformation. Strengthened “outer” connections (such as experiential and material connections) can potentially play supporting roles, but, by themselves, are unlikely to bring about transformative change. In reality, many interventions relating to strengthened connections to nature need to occur in concert, because they can be expected to interact.

### From theory to practice

Numerous practical examples exist for how types of connections between people and nature can be strengthened. Materially reconnecting people to local ecosystems can influence the parameters of a system to enhance sustainability. On a fundamental level, humanity is connected to the biosphere through the consumption of energy, goods, and other resources, but increased consumption of these is not ecologically desirable. Thus, the type of material reconnection that we advocate is a local strengthening of ties to nearby ecosystems to decouple consumption of wealthy, urban populations from impacts elsewhere in the world and increase regional self-sufficiency. Specific interventions could include restaurants serving locally grown produce, urban dwellers growing food in community gardens, or houses being built with locally sourced timber. Shortening food chains in these ways can reduce food miles with resulting benefits for CO_2_ emissions (Smith et al. 2005). Materially reconnecting to local ecosystems can also relate to other nature connections and system attributes. For example, food mile or source country labelling on products can enhance cognitive feedbacks between consumers and production landscapes. Alternatively, growing food for personal consumption can simultaneously promote sustainability, enable experiences of nature, enhance knowledge of natural processes and ecosystem functions, and contribute to emotional attachment to place (Hawkes and Acott 2013).

Many of the aforementioned material connections are closely tied to direct sustainability outcomes such as reducing carbon emissions and reducing biodiversity loss. However, these parameter changes may depend upon more fundamental systemic change. Wholesale sustainability transformation may require interventions at deep leverage points, since sustainability solutions ultimately hinge upon “value and belief systems, at levels ranging from individuals to societies” (Fischer et al. 2012a). Interventions that connect people to nature emotionally and philosophically have the greatest potential here. For example, art has the capacity to transcend the cognitive mind and convey meaning through visceral experience, and thus has considerable potential to influence the goals people pursue in life (Thomsen 2015). There is also increasing recognition of the importance of worldviews for sustainable lifestyles (Hedlund-de Witt et al. 2014). Here, the role of spirituality and religion in reorienting people towards nature is one under-researched area that has potential to function as a deep leverage point (Hitzhusen and Tucker 2013). Formal religious faiths contain teachings that promote environmental stewardship and challenge prevailing paradigms of consumption and growth (Gottlieb 2006) and can motivate action for sustainability (The Alliance of Religions and Conservation 2015). Furthermore, their spiritual practices can be powerful in shaping the deep values and beliefs people hold. Contemplative practices, such as mindfulness, even outside of a religious context are indeed powerful levers that have been found to relate to psychological nature connectedness (Howell et al. 2011) and can help promote sustainability (Wamsler et al. 2017).

Some activities that connect people with nature may simultaneously impact shallow and deep leverage points. A good example of this is community gardening. Research has shown that in addition to growing food (materially connecting to nature), allotment gardening can promote environmental learning (Bendt et al. 2013), offer therapeutic benefits (Pitt 2014), and build social cohesion and resilience (Firth et al. 2011). Similarly, nature-based education such as forest kindergartens (Waldkindergarten), popular in Germany, Sweden, and Denmark, may help Children develop deep empathy for nature in addition to developmental benefits (Kane and Kane 2011). Furthermore, interactions among forms of nature connectedness—as evident in allotment gardening or outdoor education—can offer potentially stronger leverage potential. For example, one recent study demonstrated relationships among exposure to urban nature, tree planting behaviour, and psychological connectedness to nature (Whitburn et al. 2018). Many of these initiatives are likely to be particularly powerful in urban contexts, where populations are often disconnected from experiences of nature (Miller 2005; Soga and Gaston 2016). Relating research and practice on urban greening concepts such as green infrastructure (Andersson et al. 2014), biophilic cities (Beatley 2011), and nature-based solutions (Lafortezza et al. 2017) to scholarship on sustainability transformations is, therefore, an important area for future attention in sustainability science.

Structural change may often be necessary to enable interventions for connecting people with nature to be implemented or benefits realised. For example, educational policy may need revising to allow school students’ greater interaction with nature as part of curricula, planning law may need reform to increase biological diversity within cities, and transport networks may need modification to enable people to access natural areas easily. Thus, reconnecting people with nature may both effect and depend upon deep structural change.

How interventions at deep leverage points can be scaled up is a question that sustainability scientists should actively pursue. For example, which “shallow leverage points” must be addressed in tandem for interventions at “deep leverage points” to achieve their full potential? Similarly, it is important to consider which kinds of shifts are appropriate and necessary in different social, economic, and environmental contexts. Arguably, application of the leverage point framework coupled with the typology of human–nature connections could be an effective heuristic for directing research along these lines.

## Conclusion

It is evident that reconnecting people with nature can play a useful role in addressing many of today’s ecological and sustainability challenges. To meaningfully progress a “reconnection agenda”, tangible actions must be directed towards specific changes, whether in health, education, or conservation. To this end, specifying particular types of nature connections to be enhanced is a key first step. A second step is to couch these within the literature of demonstrated causes and consequences of nature connections and a plausible theory of change (such as the concept of leverage points for sustainability transformation). Building on this theoretical foundation will enable research to move past vague speculation about the need to reconnect people with nature, and instead build an evidence base that can support research and practice.

## References

[CR1] Abson DJ, Fischer J, Leventon J (2017). Leverage points for sustainability transformation. Ambio.

[CR2] Andersson E, Barthel S, Borgström S (2014). Reconnecting cities to the biosphere: stewardship of green infrastructure and urban ecosystem services. Ambio.

[CR3] Beatley T (2011). Biophilic cities: integrating nature into urban design and planning.

[CR4] Bendt P, Barthel S, Colding J (2013). Civic greening and environmental learning in public-access community gardens in Berlin. Landsc Urban Plan.

[CR5] Bradley J, Waliczek T, Zajicek J (1999). Relationship between environmental knowledge and environmental attitude of high school students. J Environ Educ.

[CR6] Capaldi CA, Dopko RL, Zelenski JM (2014). The relationship between nature connectedness and happiness: a meta-analysis. Front Psychol.

[CR7] Collado S, Staats H, Corraliza JA (2013). Experiencing nature in children’s summer camps: affective, cognitive and behavioural consequences. J Environ Psychol.

[CR8] Conrad CC, Hilchey KG (2011). A review of citizen science and community-based environmental monitoring: issues and opportunities. Environ Monit Assess.

[CR9] Cote M, Nightingale AJ (2012). Resilience thinking meets social theory: situating social change in socio-ecological systems (SES) research. Prog Hum Geogr.

[CR10] Cumming GS, Buerkert A, Hoffmann EM (2014). Implications of agricultural transitions and urbanization for ecosystem services. Nature.

[CR12] Dorninger C, Abson DJ, Fischer J, von Wehrden H (2017). Assessing sustainable biophysical human-nature connectedness at regional scales. Env Res Lett.

[CR13] Epstein G, Vogt JM, Cox M (2013). Missing ecology: integrating ecological perspectives with the social-ecological system framework. Int J Commons.

[CR14] Ewert A, Place G, Sibthorp J (2005). Early-life outdoor experiences and an individual’s environmental attitudes. Leis Sci.

[CR15] Firth C, Maye D, Pearson D (2011). Developing “community” in community gardens. Local Environ.

[CR16] Fischer J, Dyball R, Fazey I (2012). Human behavior and sustainability. Front Ecol Environ.

[CR17] Fischer J, Hartel T, Kuemmerle T (2012). Conservation policy in traditional farming landscapes. Conserv Lett.

[CR18] Fischer J, Gardner TA, Bennett EM (2015). Advancing sustainability through mainstreaming a social–ecological systems perspective. Curr Opin Environ Sustain.

[CR19] Folke C, Jansson Å, Rockström J (2011). Reconnecting to the biosphere. Ambio.

[CR20] Frantz CM, Mayer FS (2014). The importance of connection to nature in assessing environmental education programs. Stud Educ Eval.

[CR21] Geng L, Xu J, Ye L (2015). Connections with nature and environmental behaviors. PLoS One.

[CR22] Gosling E, Williams KJH (2010). Connectedness to nature, place attachment and conservation behaviour: Testing connectedness theory among farmers. J Environ Psychol.

[CR23] Gottlieb RS (2006) Introduction. In: The oxford handbook of religion and ecology. Oxford University Press, Oxford, pp 3–21. http://www.oxfordhandbooks.com/view/10.1093/oxfordhb/9780195178722.001.0001/oxfordhb-9780195178722

[CR24] Haberl H, Fischer-Kowalski M, Krausmann F (2004). Progress towards sustainability? What the conceptual framework of material and energy flow accounting (MEFA) can offer. Land use policy.

[CR25] Haberl H, Erb K-H, Krausmann F (2009). Using embodied HANPP to analyze teleconnections in the global land system: conceptual considerations. Geogr Tidsskr J Geogr.

[CR26] Hawkes FM, Acott TG (2013). People, environment and place: The function and significance of human hybrid relationships at an allotment in South East England. Local Environ.

[CR27] Hedlund-de Witt A, de Boer J, Boersema JJ (2014). Exploring inner and outer worlds: a quantitative study of worldviews, environmental attitudes, and sustainable lifestyles. J Environ Psychol.

[CR28] Hitzhusen GE, Tucker ME (2013). The potential of religion for Earth Stewardship. Front Ecol Environ.

[CR29] Howell AJ, Dopko RL, Passmore H-A, Buro K (2011). Nature connectedness: associations with well-being and mindfulness. Pers Individ Dif.

[CR30] Ives CD, Giusti M, Fischer J (2017). Human-nature connection: a multidisciplinary review. Curr Opin Environ Sustain.

[CR31] Kahn PHJ, Kellert SR (2002). Children and nature: psychological, sociocultural, and evolutionary investigations.

[CR32] Kals E, Schumacher D, Montada L (1999). Emotional affinity toward nature as a motivational basis to protect nature. Environ Behav.

[CR33] Kane BA, Kane J (2011). Waldkindergarten in Germany. Green Teach.

[CR01] Kareiva P (2008). Ominous trends in nature recreation. Proc Natl Acad Sci.

[CR34] Keniger LE, Gaston KJ, Irvine KN, Fuller RA (2013). What are the Benefits of Interacting with Nature?. Int J Environ Res Public Health.

[CR35] Lafortezza R, Chen J, van den Bosch CK, Randrup TB (2017). Nature-based solutions for resilient landscapes and cities. Environ Res doi.

[CR36] Lin BB, Fuller RA, Bush R (2014). Opportunity or orientation? Who uses urban parks and why. PLoS One.

[CR37] Liu J, Hull V, Batistella M (2013). Framing sustainability in a telecoupled world. Ecol Soc.

[CR02] Louv R (2005). Last child in the woods: saving our children from nature-deficit disorder.

[CR38] Lumber R, Richardson M, Sheffield D (2017) Beyond knowing nature: contact, emotion, compassion, meaning, and beauty are pathways to nature connection. PLoS One 1–24 10.1371/journal.pone.0177186 PMC5423657 28486515

[CR39] Mainella FP, Agate JR, Clark BS (2011). Outdoor-based play and reconnection to nature: a neglected pathway to positive youth development. New Dir Youth Dev.

[CR40] Mayer FS, Frantz CM (2004). The connectedness to nature scale: A measure of individuals’ feeling in community with nature. J Environ Psychol.

[CR41] Meadows D (1999). Leverage points: places to intervene in a system.

[CR42] Millenium Ecosystem Assessment (2003). Ecosystems and human well-being: a framework for assessment.

[CR43] Miller JR (2005). Biodiversity conservation and the extinction of experience. Trends Ecol Evol.

[CR44] Muhar A, Raymond CM, Van den Born RJG (2017). A model integrating social-cultural concepts of nature into frameworks of interaction between social and natural systems. J Environ Plan Manag.

[CR45] Nisbet EK, Zelenski JM, Murphy SA (2009). The nature relatedness scale: linking individuals’ connection with nature to environmental concern and behavior. Environ Behav.

[CR46] Pergams ORW, Zaradic PA (2006). Is love of nature in the US becoming love of electronic media? 16-year downtrend in national park visits explained by watching movies, playing video games, internet use, and oil prices. J Environ Manage.

[CR47] Pitt H (2014). Therapeutic experiences of community gardens: Putting flow in its place. Heal Place.

[CR48] Pyle RM (1993). The thunder tree: lessons from an urban wildland.

[CR49] Pyle RM (2003). Nature matrix: reconnecting people and nature. Oryx.

[CR50] Raymond CM, Singh GG, Benessaiah K (2013). Ecosystem services and beyond: using multiple metaphors to understand human-environment relationships. Bioscience.

[CR51] Restall B, Conrad E (2015). A literature review of connectedness to nature and its potential for environmental management. J Environ Manage.

[CR52] Schroeter M, van der Zanden EH, van Oudenhoven APE (2014). Ecosystem services as a contested concept: a synthesis of critique and counter-arguments. Conserv Lett.

[CR53] Schultz PW (2001). The structure of environmental concern: concern for self, other people, and the biosphere. J Environ Psychol.

[CR54] Seppelt R, Cumming GS (2016). Humanity’s distance to nature: time for environmental austerity?. Landsc Ecol.

[CR55] Shanahan DF, Bush R, Gaston KJ (2016). Health benefits from nature experiences depend on dose. Sci Rep.

[CR56] Silvertown J (2015). Have ecosystem services been oversold?. Trends Ecol Evol.

[CR11] Smith A, Watkiss P, Tweddle G, McKinnon AC (2005). The validity of food miles as an indicator of sustainable development: final report for DEFRA.

[CR57] Soga M, Gaston KJ (2016). Extinction of experience: the loss of human-nature interactions. Front Ecol Environ.

[CR58] Stedman RC (2003). Is it really just a social construction? The contribution of the physical environment to sense of place. Soc Nat Resour.

[CR59] Steffen W, Persson A, Deutsch L (2011). The anthropocene: From global change to planetary stewardship. Ambio.

[CR60] Tam KP (2013). Concepts and measures related to connection to nature: Similarities and differences. J Environ Psychol.

[CR61] Taniguchi ST, Freeman PA, Richards AL (2005). Attributes of meaningful learning experiences in an outdoor education program. J Adventure Educ Outdoor Learn.

[CR62] The Alliance of Religions and Conservation (2015) Faith in the future: the Bristol Commitments. http://arcworld.org/downloads/Faith-in-the-Future-with-cover(UN).pdf. Accessed 18 Jan 2018

[CR63] Thomsen DC (2015). Seeing is questioning: prompting sustainability discourses through an evocative visual agenda. Ecol Soc.

[CR64] Townsend M (2006). Feel blue? Touch green! Participation in forest/woodland management as a treatment for depression. Urban For Urban Green.

[CR65] UNEP; Fischer-Kowalski M, Swilling M, von Weizsäcker EU, Ren Y, Moriguchi Y, Crane W, Krausmann F, Eisenmenger N, Giljum S, Hennicke P, Romero Lankao P, Siriban Manalang A (2011) Decoupling natural resource use and environmental impacts from economic growth, A Report of the Working Group on Decoupling to the International Resource Panel. Available online: http://www.gci.org.uk/Documents/Decoupling_Report_English.pdf. Accessed 21 Feb 2018

[CR66] Valentine G, McKendrck J (1997). Children’s outdoor play: Exploring parental concerns about children’s safety and the changing nature of childhood. Geoforum.

[CR67] Van den Born RJG (2008). Rethinking nature: public visions in the Netherlands. Environ Values.

[CR68] Wackernagel M, Onisto L, Bello P (1999). National natural capital accounting with the ecological footprint concept. Ecol Econ.

[CR69] Wamsler C, Brossmann J, Hendersson H (2017). Mindfulness in sustainability science, practice, and teaching. Sustain Sci.

[CR70] Whitburn J, Linklater WL, Milfont TL (2018). Exposure to urban nature and tree planting are related to pro-environmental behavior via connection to nature, the use of nature for psychological restoration, and environmental attitudes. Environ Behav.

[CR71] Wilson EO (1984). Biophilia: the human bond with other species.

[CR72] Yu Y, Feng K, Hubacek K (2013). Tele-connecting local consumption to global land use. Glob Environ Chang.

[CR73] Zaradic PA, Pergams ORW, Kareiva P (2009). The impact of nature experience on willingness to support conservation. PLoS One.

[CR74] Zylstra MJ, Knight AT, Esler KJ, Le Grange LLL (2014). Connectedness as a core conservation concern: an interdisciplinary review of theory and a call for practice. Springer Sci Rev.

